# Determination of orally administered 1,8-Cineol in nasal polyp tissues from chronic rhinosinusitis patients using gas chromatography: mass spectrometry

**DOI:** 10.1038/s41598-023-29941-x

**Published:** 2023-03-03

**Authors:** Claire MacKenzie, Thomas Goerke, Mark Buecking, Mathias Heidemann, Anke Leichtle, Benedikt Ringbeck, Friederike Möllenkolk, Michael Ploch, Karl-Ludwig Bruchhage, Ralph Pries

**Affiliations:** 1 Fraunhofer Institute for Molecular Biology and Applied Ecology IME, Schmallenberg, Germany; 2 Klosterfrau Healthcare Group, Berlin, Germany; 3 Department of Otorhinolaryngology, University Hospital of Schleswig-Holstein, Campus Lübeck, Ratzeburger Allee 160, 23538 Lübeck, Germany

**Keywords:** Drug discovery, Molecular biology, Diseases, Health care, Medical research

## Abstract

Chronic rhinosinusitis with nasal polyps (CRSwNP) is a common inflammatory disease causing considerable disease burden. The anti-inflammatory monoterpene 1,8-Cineol is a natural plant-based therapeutic agent that is well established to treat chronic and acute airway diseases. Aim of this study was to investigate whether the herbal drug 1,8-Cineol reaches the nasal tissue via the gut and the blood stream upon its oral administration. A highly sensitive gas chromatography mass spectrometry-based method with stir bar sorptive extraction (SBSE) for sample preparation has been developed and validated for the extraction, detection and quantification of 1,8-Cineol in tissue samples of nasal polyps from 30 CRSwNP patients. Data revealed a highly sensitive detection of 1,8-Cineol in nasal tissue samples after 14 days of oral administration of 1,8-Cineol prior to surgical treatment. There was no significant correlation between the measured 1,8-Cineol concentrations and bodyweight or BMI values of the analyzed patients, respectively. Our data indicate a systemic distribution of 1,8-Cineol in the human body after its oral administration. Individual differences in terms of metabolic characteristics and have to be further investigated. The study increases our understanding of the systemic effects of 1,8-Cineol upon its therapeutic application and benefit in patients with CRSwNP.

## Introduction

Chronic rhinosinusitis with nasal polyps (CRSwNP) is a common disease worldwide and affecting about 10% of the European population^1,2^. CRSwNP is frequently associated with asthma and allergic rhinitis but the cellular and molecular mechanisms that contribute to the clinical symptoms are not fully understood. Although the pathogenesis and inflammatory processes of this disease have been extensively studied, the exact multifactorial mechanisms still remain elusive^3^. Various factors are known to be associated with CRSwNP, such as air pollution, individual immune barrier dysfunctions or alterations in the eicosanoid pathway^4–7^. Furthermore, microbial pathogens such as *Staphylococcus aureus* are suspected as a trigger for CRSwNP^8,9^. Most established therapies of CRSwNP comprise the administration of corticosteroids or antibiotics and surgery^10^. Furthermore, different biologicals targeting immunoglobulin E (IgE), as well as interleukins (IL) IL-5, IL-4, and IL-13 have been introduced recently^11,12^.

The anti-inflammatory monoterpene 1,8-Cineol (1,3,3-trimethyl-2-oxabicyclo[2.2.2]octane) is a natural plant-based therapeutic agent that is commonly applied to treat various chronic and acute airway diseases as well as patients with CRSwNP. The eucalyptus tree (*Eucalyptus spec.*) represents the major natural source of 1,8-Cineol, but other plants such as oregano (*Origanum spec.),* thyme (*Thymus spec.*), or sage (*Salvia spec.*) contain this secondary plant metabolite as well^13–15^. 1,8-Cineol is increasingly perceived as a non-prescription mucolytic medication in inflammatory diseases such as bronchitis or chronic obstructive pulmonary disease (COPD)^16–18^. In addition, 1,8-Cineol containing eucalyptus oil was shown to decrease allergic reactions by suppressing the degranulation of mast cells^19^.

1,8-Cineol is a colorless and liquid lipophilic with a density of 0.93 g/cm^3^ (at 20 °C) and camphor-like aroma. The molecular formula of Cineol is C_10_H_18_O with a molecular weight of 154.25 g/mol^20^.

1,8-Cineol leads to significantly reduced expression of pro-inflammatory mediators such as TNF-α, IL-1β, and IL-6 from monocytes^21,22^ as well as the IL-4 and IL-5 production from lymphocytes^23^. An anti-microbial activity of 1,8-Cineol has been described in different inflammatory diseases^16,24–26^ and cytotoxic effects on cancer cells are also evident^27,28^. However, the systemic distribution of 1,8-Cineol in the human body and the associated direct and indirect therapeutic effects are not understood so far.

It has been shown that the metabolization of 1,8-Cineol is maintained by the cytochrome P450 system in the mammalian liver^29,30^. Accordingly, various Cineol related metabolites (2-hydroxy-, 3-hydroxy-, 7-hydroxy- and 9-hydroxy-1,8-Cineol) have been identified in human plasma or urine samples after oral application of 1,8-Cineol^31^. In an earlier study the pharmacokinetics of 1,8-Cineol during prolonged inhalation have been investigated. The results showed that 1,8-Cineol was well absorbed from breathing air and detectable in the blood plasma after approximately 18 min, with a mean distribution half-life of about 7 min^32^.

Thus, the crucial question is, if 1,8-Cineol is applied orally, does it act indirectly via a systemic immunomodulation via the gut or the blood stream or does it act as well directly anti-inflammatory and bactericidal on site in the nasal epithelium?

## Results

### Quantification of 1,8-Cineol using GC–MS

**Figure 1:**
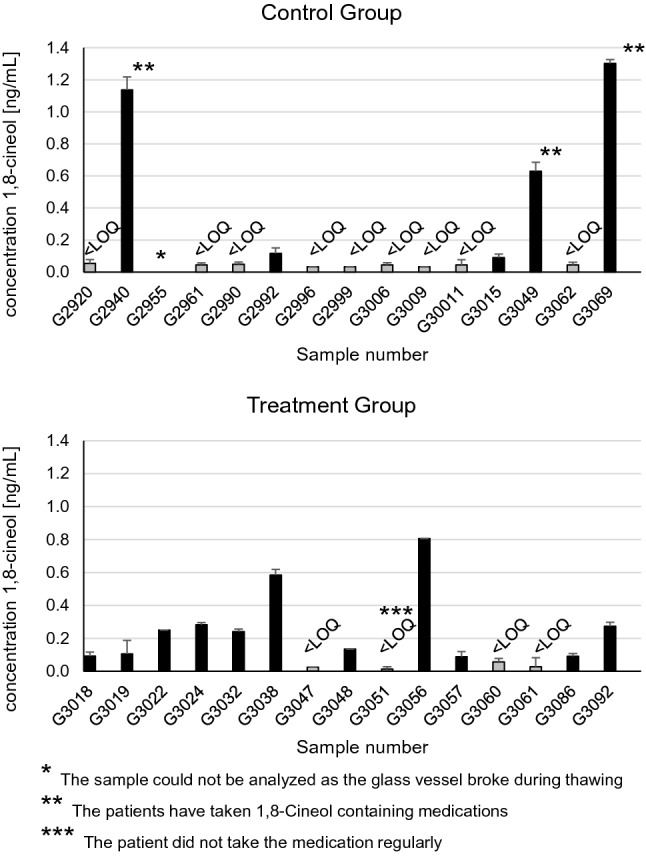
Concentrations of 1,8-Cineol in tissue extracts of nasal polyps from CRSwNP patients after 14 days of oral 1,8-Cineol administration. Results are shown as mean values of triplicates and confidence intervals. Concentrations below LOQ were set to ½*LOQ (gray bars). Concentrations above LOQ are shown as black bars. Sample G3024 was measured in duplicate only as the PDMS twister broke.

We analyzed the concentration of 1,8-Cineol in tissue samples of nasal polyps from 30 CRSwNP patients, whereby 15 patients received an oral administration of 1,8-Cineol for 14 days prior surgical treatment (treatment group). 1,8-Cineol was detected above the LOQ in 11 out of 15 analyzed tissue samples from the 1,8-Cineol receiving patient cohort (Fig. 1). In the samples of the control group, 1,8-Cineol could be detected above the LOQ in 5 out of 15 samples. Surprisingly, the data revealed remarkably high levels of 1,8-Cineol in tissue samples of three patients from the control group (G2940; G3049; G3069) (Fig. 1).

In the analyzed tissue samples of the treatment group our data revealed large individual differences in terms of the detected 1,8-Cineol concentrations, whereas samples of 4 patients were below the quantification limit.

**Figure 2:**
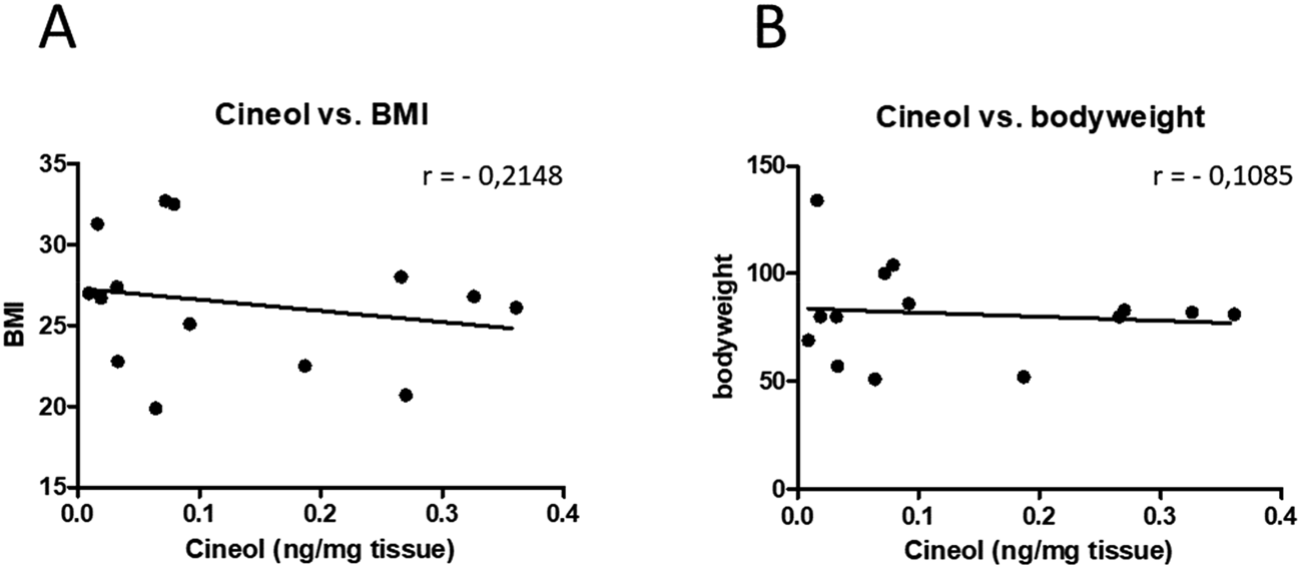
Correlation analysis between measured 1,8-Cineol concentrations (ng 1,8-Cineol /mg tissue) and patients (**A**) body mass index (BMI) and (**B**) bodyweight values. The Pearson correlation coefficient (r) is given.

Correlation analysis of the CRSwNP patient cohort between measured 1,8-Cineol concentrations in the analyzed tissue samples (ng 1,8-Cineol/mg tissue) and bodyweight and BMI values revealed no significant correlations (Fig. 2).

**Figure 3:**
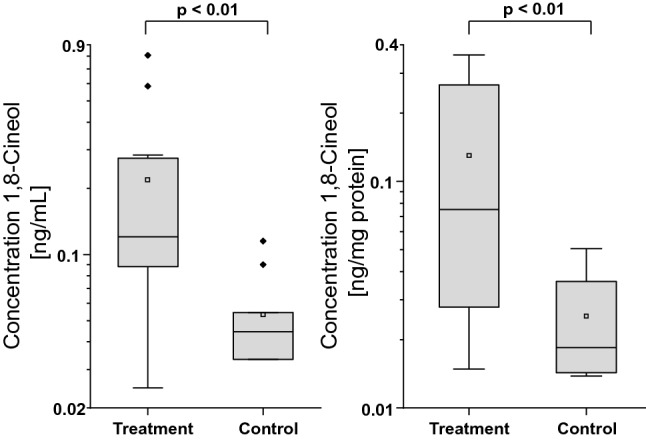
Concentrations of 1,8-Cineol in ng/mL and ng/mg protein of the treatment group and control group shown as boxplots. Significant differences were calculated by Mann–Whitney-U test and results are shown as p values. Concentrations below LOQ were set to ½*LOQ. Samples from control patients who used 1,8-Cineol medications contrary to agreement and samples from the treatment group who did not use the 1,8-Cineol medication regularly, were excluded from statistical analysis.

In the treatment group, 1,8-Cineol concentrations were significantly (p < 0.01) higher than in the control group, both for concentrations in ng/mL lysate and ng/mg protein, showing that 1,8-Cineol is distributed into, i.e., nasal tissues after oral uptake. There was no correlation found between 1,8-Cineol concentration and the protein concentration in the lysate (Pearson correlation coefficient r = 0.07; p > 0.05 (Fig. 3).

## Discussion

The present study revealed new insights into the distribution of 1,8-Cineol in the human body after oral administration and hint to a direct anti-inflammatory effect of 1,8-Cineol on site in nasal polyps of CRSwNP patients. The bioactive effects of 1,8-Cineol are known to be limited due to its low aqueous solubility and stability^33^. Different well-known 1,8-Cineol containing medications are applied orally as enteric coated capsules and evolve their curative effects after their passage through the stomach within the small intestine. Therefore, our aim was to investigate whether a systemic distribution of 1,8-Cineol leads to an accumulation in nasal polyp tissues from CRSwNP patients after a two-week 1,8-Cineol oral administration prior to surgical treatment.

It has recently been shown in a rat pharmacokinetic study, that serum concentration–time profiles of 1,8-Cineol after an oral administration were similar compared to an intravenous administration, which underlines the systemic distribution of 1,8-Cineol^34^. In our study, 1,8-Cineol was clearly detectable in tissue samples of nasal polyps, independently from the individual bodyweight or BMI values of the patients. Oral administration of 1,8-Cineol containing medicine led to significantly increased 1,8-Cineol concentrations in nasal polyps, which is a basis for a potential local therapeutic effect. We identified strong individual deviations of detected 1,8-Cineol concentrations in nasal polyp tissues after two-weeks oral administration, which are most likely due to individual metabolic characteristics in terms of systemic uptake of 1,8-Cineol via the small intestine or its metabolism in the liver, respectively. 1,8-Cineol concentrations were positively skewed, while protein concentrations in the tissues were normally distributed. Further surveys of three patients revealed an individual unsolicited application of alternative 1,8-Cineol containing essential oils (G3069: capsules; G2940: nasal spray; G3049: inhalation). Two samples (G2992; G3015) had concentrations just slightly above LOQ which might be explained by uptake of 1,8-Cineol from natural sources such as herbs^27–29^.

Further deviations are probably due to an individual irregular medication intake or individual bodyweight and body mass index (BMI) values, respectively. Accompanying patient surveys revealed that, against the agreement, patient G3051 indeed did not regularly take the medication, whereas the other three patients (G3047; G3060; G3061) stated that they took the medication regularly throughout the period of 14 days. Our data revealed no correlation between 1,8-Cineol concentration and the protein concentration in the lysate, which indicates that 1,8-Cineol is not bound to nasal polyp proteins and therefore might be bioavailable in the cells.

The biotransformation of 1,8-Cineol has been analyzed in vitro using human liver microsomes as well as recombinant cytochrome P450 enzymes, indicating a clear correlation between the concentration of the metabolites, incubation time and enzyme content^29^. Further studies need to be performed concerning the distribution and metabolism of 1,8-Cineol in terms of novel clinical administration approaches and the underlying immunological interplay in profiting patient cohorts.

## Methods

### Ethics statement

Medical examinations and surgical treatments were carried out at the Department of Otorhinolaryngology, University Hospital Schleswig–Holstein, Campus Lübeck. All patients have given their written informed consent. The study was approved by the local ethics committee of the University of Lübeck (approval number 18–322) and conducted in accordance with the ethical principles for medical research formulated in the WMA Declaration of Helsinki.

### Tissue samples

Patients were recruited to the study in the period from June to October 2020 based on the diagnostic criteria of CRS including medical history, physical examination, nasal endoscopy, and computed tomography (CT) scan of the sinuses. Nasal polyp tissue specimens were obtained laterally to the concha nasalis media during endonasal sinus surgery from CRSwNP patients (n = 15) after 14 days of 1,8-Cineol administration as well as from CRSwNP patients (n = 15) without prior 1,8-Cineol administration. Before surgery, all patients had been free of steroid medication for at least four weeks. Fresh tissue samples were flash frozen in liquid nitrogen immediately after resection and stored at − 80 °C.

1,8-Cineol (CNL-1976) was used in terms of the clinically approved drug Soledum^®^ Kapseln forte (capsules) (Cassella-med GmbH & Co. KG, Cologne, Germany). For therapeutic use patients have been prescribed Soledum capsules (3 × 200 mg/day) over 14 days. We analyzed 30 CRSwNP patients (22 men, 8 women) with a mean age of 50.9 years.

Tissue homogenates of nasal polyps from 30 CRSwNP patients were prepared in glass vials using the Omni Tissue Master Homogenizer (Perkin Elmer GmbH, Hamburg, Germany) in ice cold phosphate buffered saline (PBS). The protein concentrations of the samples were determined using Bradford's assay (BioRad Laboratories GmbH, Munich, Germany) following the manufacturer's instructions. Samples were stored at − 80 °C.

### Sample extraction and analysis

For analysis, samples were shipped to Fraunhofer Institute for Molecular Biology and Applied Ecology (IME) on dry ice at − 80 °C. Samples were thawed in a refrigerator at 4 °C and subsequently brought to room temperature. Afterwards, samples were homogenized by shaking on a Vortex mixer and an aliquot of 1.5 mL was added to 2.5 mL of distilled water. If too little sample was available, the volume of the aliquot was reduced accordingly and made up to a total volume of 4 mL with distilled water. A clean polydimethylsiloxane (PDMS) coated magnetic stir bar (Twister^®^, Gerstel GmbH & Co. KG, Mülheim an der Ruhr, Germany) was used each to extract the 1,8-Cineol from the samples. 5 µL of the internal standard 1,4-Cineol was added to the solution. Samples were extracted at 20 °C for 1.5 h at a setting of 500 revolutions per minute (rpm) on a magnetic stirrer. The Twister was shortly dried under nitrogen flow and placed into a fitted glass tube ready for thermal desorption and subsequent GC–MS analysis. Three aliquots of each sample were analyzed, except for one sample from the control batch (G29255) where the glass vial broke during thawing and accordingly, no sample could be taken for analysis. Furthermore, in one aliquot of a sample from the treatment group (G3024) the twister had a defect and therefore only two aliquots could be analyzed. In a few aliquots, less than 1.5 mL could be extracted, however, this is considered in the calculation. The average volume of samples that could be extracted was 1.43 mL and was the same in both the control and treatment group, so it does not negatively influence the statistical evaluation.

The tubes containing the Twisters were transferred to the thermal desorption unit using an MPS 2 autosampler system (Gerstel GmbH & Co. KG) and desorbed with the temperature program and described below at a transfer temperature of 260 °C in splitless mode. The unit started at 30 °C, after 0.25 min it was heated to 250 °C with a rate of 360 °C/min. The temperature was kept for 5 min to allow desorption of the analytes from the Twister. The analyte was trapped before the chromatographic column in a cold injection system (CIS) with liquid nitrogen cryo-cooling on a CIS4 glass liner (baffled, not activated) (Gerstel GmbH & Co. KG) under conditions and following temperature program described below. The system was cooled to − 50 °C during the time of thermal desorption to trap the analytes in the unit. For sample introduction to the GC the system was kept at − 50 °C for 0.1 min and heated to 260 °C with a rate of 12 °C/s. The temperature was held for 3 further minutes to allow complete introduction to the GC system.

### Gas chromatography—mass spectrometry

A sensitive gas chromatography-mass spectrometry (GC–MS) method has been developed and validated for the detection and quantification of 1,8-Cineol in tissue samples of nasal polyps from CRSwNP patients.

The analytes were chromatographically separated on a fused silica column with 95% dimethyl and 5% diphenyl polysiloxane (Rtx-5 Amine; 30 m, 0,25 mm ID, 0,25 µm film-thickness; Restek Corporation, Bellefonte, PA, USA) using a 6890N with MSD 5973 Network gas chromatography mass spectrometry system (Agilent Technologies Inc., Santa Clara, CA, USA). The temperature program used for chromatographic separation is described in Table 1. Helium (5.0, Messer GmbH, Bad Soden am Taunus, Germany) at a constant flow of 1.5 mL/min was used as a carrier gas. Electron ionization (EI) at 70 eV was used at a source temperature of 230 °C. Mass spectrometric determination was performed in selective ion monitoring (SIM) mode, analyzing the masses *m/z* 154 (used for quantification) and *m/z* 108 as a qualifier for 1,8-Cineol. Quantifier and qualifier mass transitions for 1,4-Cineol were *m/z* 111 and *m/z* 154, respectively. MS quadrupole temperature was 150 °C. A solvent delay of 4 min was used with following acquisition for 10 min.

**Table 1:** Temperature program of the gas chromatograph.

Ramp no	Rate [°C/min]	End temperature [°C]	Time [min]
Start	0	50	2
1	20	130	0
2	30	250	0
Post-run	0	290	2

MassHunter GC/MS Acquisition (B.07.06.2704, Agilent Technologies. Inc. Santa Clara, CA, USA) and MassHunter Workstation Quantitative Analysis (B.09.00) were employed for data acquisition and evaluation.

### Method validation and quality assurance

**Figure 4:**
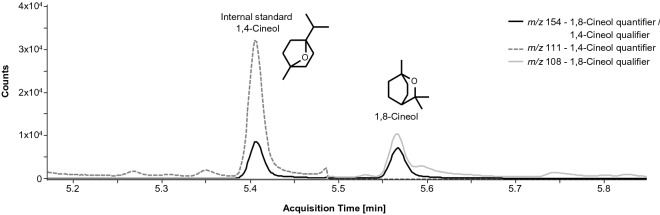
Representative chromatogram of the test sample G3038 (treatment group), replicate A, including the quantifier and qualifier mass transitions of 1,8-Cineol and the internal standard 1,4-Cineol and chemical structures of both analytes.

The method was validated in terms of selectivity, recovery and reproducibility. All experiments including validation and analysis of samples were conducted under Good Laboratory Practice (GLP). The limit of quantification (LOQ) was set at 0.1 ng/sample extracted from cell lysate independent of the sample volume or mass. The limit of detection (LOD) was set at 0.05 ng/sample (½ LOQ). The validated linear weighted (concentration^−1^) calibration range covers concentrations from 0.05 to 5 ng/sample. Coefficients of determination were ≥ 0.999. An exemplary chromatogram of a test sample is shown in Fig. 4.

Reproducibility was checked at LOQ level with 6 individual samples. A mean recovery of 102% with a relative standard deviation of 2.7% was achieved. Three blank samples containing only water and 3 samples containing cell lysate were analyzed to ensure specificity of the method. All blank samples showed no concentration above LOD. Further samples containing matrix were fortified at 0.1 ng/sample, 1.0 ng/sample, and 4.0 ng/sample in triplicate. Mean recoveries were 121% with a relative standard deviation (RSD) of 9.09%, 107% with an RSD of 4.12%, and 98.7% with an RSD of 0.181%, respectively, proving the accuracy and precision of this method.

Quality control samples were analyzed with each sample batch to ensure the applicability of the calibration and performance of the system. They were prepared in the same manner as the validation samples at 1.0 ng/sample and 3.75 ng/sample.

### Statistical analysis

Statistical analyses for associations with BMI and body weight were performed with GraphPad Prism Version 7.0f. The mean and standard error (SEM) are presented. The correlation between parameters was calculated using multivariate regression with the Pearson correlation coefficient. Shapiro–Wilk test was used to test normality. Accordingly, the non-parametric Mann–Whitney-U test was used to test for significant differences between groups. Boxplots were prepared and further statistical analyses (Shapiro–Wilk test, Mann–Whitney-U test, and Pearson correlation between 1,8-Cineol concentration and protein concentration) were conducted with OriginPro 2021 ((9.8.0.200), OriginLab Corporation, Northhampton, Ma, USA). Sample concentrations below LOQ were set to ½*LOQ for further calculation. Number of samples and additional statistical details are given in the respective figure legends, when appropriate.

## Data Availability

The data presented in this study are available on request from the corresponding author.
